# The Physician Himself

**Published:** 1885-05

**Authors:** 


THE PHYSICIAN HIMSELF, AND WHAT HE SHOULD ADD TO HIS SCIENTIFIC ACQUIREMENTS IN ORDER TO SECURE SUCCESS. By D. W. Cathell, M. D., late Professor of Pathology in the College of Physicians and Surgeons of Baltimore. Fourth Edition. Baltimore: Cushings & Bailey, 226 W. Baltimore street. 1885.

In presenting this carefully revised edition, a great many new suggestions have been added by the author, and instead of the clap-trap measures too often resorted to by novices in the medical profession, he has given the true landmarks by which the practitioner should be guided in his intercourse with his patients and his colleagues.

A proper regard for the true dignity of our calling and our self-respect should be kept prominently before the members of our profession, and this essay on *Personal Questions in Medical Practice* is calculated to subserve this end.

